# Care for Patients With Neuromuscular Disorders in the COVID-19 Pandemic Era

**DOI:** 10.3389/fneur.2021.607790

**Published:** 2021-03-24

**Authors:** Yung-Hao Tseng, Tai-Heng Chen

**Affiliations:** ^1^Department of Pediatrics, Division of Pediatric Emergency, Kaohsiung Medical University Hospital, Kaohsiung Medical University, Kaohsiung, Taiwan; ^2^School of Post-Baccalaureate Medicine, College of Medicine, Kaohsiung Medical University, Kaohsiung, Taiwan

**Keywords:** neuromuscular disorder, multidisciplinary care, respiratory care, COVID- 19, telemedicine

## Abstract

The coronavirus disease 2019 (COVID-19) pandemic has prompted a rapid and unprecedented reorganization of medical institutions, affecting clinical care for patients with chronic neurological diseases. Although there is no evidence that patients with neuromuscular disorders (NMD) confer a higher infection risk of COVID-19, NMD and its associated therapies may affect the patient's ability to cope with infection or its systemic effects. Moreover, there is a concern that patients with chronic NMD may be at increased risk of manifesting severe symptoms of COVID-19. In particular, as respiratory compromises account for the major cause of mortality and morbidity in NMD patients, newly emerging data also show that the risk of exacerbation caused by COVID-19 accumulates in this particular patient group. For example, patients with motor neuron disease and dystrophinopathies often have ventilatory muscle weakness or cardiomyopathy, which may increase the risk of severe COVID-19 infection. Thus, the COVID-19 pandemic may severely affect NMD patients. Several neurological associations and neuromuscular networks have recently guided the impact of COVID-19 on patients with NMD, especially in managing cardiopulmonary involvements. It is recommended that patients with moderate- to high-risk NMD be sophisticatedly monitored to reduce the risk of rapid decline in cardiopulmonary function or potential deterioration of the underlying NMD. However, limited neuromuscular-specific recommendations for NMD patients who contract COVID-19 and outcome data are lacking. There is an urgent need to properly modify the respiratory care method for NMD patients, especially during the COVID-19 pandemic. Conclusively, COVID-19 is a rapidly evolving field, and the practical guidelines for the management of NMD patients are frequently revised. There must be a close collaboration in a multidisciplinary care team that should support their hospital to define a standardized care method for NMD patients during the COVID pandemic. This article reviews evidence-based practical guidelines regarding care delivery, modification, and education, highlighting the need for team-based and interspecialty collaboration.

## Introduction

In December 2019, a severe pneumonia outbreak related to a novel coronavirus disease 2019 (COVID-19) began in Wuhan, China, and soon spread across the world. Compared with severe acute respiratory syndrome coronavirus-2 (SARS-CoV-2) that caused a SARS epidemic in 2003, COVID-19 has a more robust transmission capacity, making prevention and control more complex. As of September 14, 2020, COVID-19 has caused more than 28 million infections, including more than 900,000 deaths worldwide (1). In a short period, the pandemic has greatly changed the current guidelines for managing patients with chronic neurological diseases, leading to a significant impact on the field. This pandemic urges a rapid and unprecedented readjustment of medical services, especially in patients with neuromuscular disease (NMD) known to have an increased risk of severe COVID-19 disease course. Overall, NMD constitutes a group of heterogeneous diseases, most of which are of genetic or autoimmune origins that affect individuals of all ages. The categories of NMD usually, but not exclusively, include muscle disorders (e.g., congenital muscular dystrophies, myopathies, and muscle channelopathies), motor neuron disorders [e.g., spinal muscular atrophy, spinal muscular atrophy (SMA), and amyotrophic lateral sclerosis (ALS)], diseases of the neuromuscular junction [e.g., myasthenia gravis (MG) and Lambert–Eaton myasthenic syndrome], and peripheral nerve disorders (e.g., inflammatory demyelinating polyneuropathy and hereditary motor and sensory neuropathy). Many patients with NMD exhibit multiple disabilities and usually have cardiopulmonary complications. However, since most NMD categories cover various diagnoses and degrees of severity, it is difficult to make specific recommendations that are generally applicable even among patients with the same diagnosis.

So far, there is no evidence that hereditary NMD will increase the risk of SARS-CoV-2 infection; however, comorbidities associated with NMD and its treatment may affect patients' ability to cope with COVID-19 (2–4). Therefore, risk identification and stratification are essential to assess NMD patients' susceptibility to developing a serious course of COVID-19. Following the guidance of the Britain National Neurological Associations and neuromuscular networks, the World Muscle Society announced its position and recommendations regarding the influence of COVID-19 on NMD and associated management (2). These guidances recognize the risk of severe COVID-19 disease course as high or moderately high in all but the mildest forms of NMD. The risk is significantly increased in NMD patients associated with certain comorbidities (Table 1). For example, factors that may confer increased risks of severe prognosis in NMD patients should they be infected with COVID-19 include respiratory compromises, myocardial impairment, or using immunosuppressive medications. Moreover, several additional risk factors might further exacerbate the pre-existing debilitation and increase infectious risk in susceptible patients with NMD (5, 6).

**Table 1:** Features of NMD patients conferring higher risk of severe COVID-19 infection.

**Affected aspect**	**Associated features**	**Susceptible NMD types**
Respiratory system	•Weakness of respiratory muscles or diaphragm, resulting in respiratory volumes <60% predicted (FVC<60%) •Use of ventilation *via* mask or tracheotomy •Weak cough and weak airway clearance due to oropharyngeal weakness (bulbar involvement) •Presence of tracheostoma	Any kinds of NMD with respiratory muscle involvement, especially severe -to-moderate types of SMA, ALS, end-stage DMD, severe congenital myopathies, and congenital muscular dystrophies
Cardiac system	NMD-related cardiomyopathy, conductive arrhythmias, and/or on medications for cardiac involvement	DMD/BMD, Emery-Dreifuss muscular dystrophy, facioscapulohumeral muscular dystrophy (especially infantile form)
Systemic involvement	Risk of deterioration with fever, fasting or infection	Mitochondrial myopathies, metabolic myopathies, SMA
	Risk of rhabdomyolysis with fever, fasting or infection	Mitochondrial myopathies, metabolic myopathies
	Concomitant diabetes and obesity	NMD with inborn metabolic disorders
Medication History	Patients taking steroids and undergoing immunosuppressant treatment	Inflammatory myopathies (e.g., polymyositis, dermatomyositis), DMD/BMD, myasthenia gravis, congenital myasthenic syndrome
Additional risk factors	•Kyphoscoliosis •Highly-active immune-mediated NMD •Older age •Pregnancy (possible) •Concomitant additional neurologic diseases •Dependence from caregivers in hygiene, mobilization and feeding	Any kinds of NMD with associated risk factors

*NMD, neuromuscular disorder; FVC, forced vital capacity; SMA, spinal muscular atrophy; ALS, amyotrophic lateral sclerosis; DMD, Duchenne muscular dystrophy; BMD, Becker muscular dystrophy*.

## Practical Guidance for NMD Patients in the Covid-19 Pandemic

The COVID-19 pandemic has prompted the rapid reorganization of hospital settings and patient service provision to cope with emerging but unmet medical needs. In particular, the prevention strategies produce impacts on the management for patients with NMD (4, 6). Patients should ensure that they have sufficient medication (at least 1 month) and ventilatory support equipment (2). Switching to patient appointments for telephone interviews helps eliminate the risk of contracting COVID-19. Patients and caregivers should know how to utilize online and telephone-based pharmacies, equipment ordering, and delivery services (7). Nevertheless, social distancing remains the most important intervention to limit the spread of COVID-19, and if possible, all NMD patients should wear masks upon their arrival at the hospital (8).

### Management of Immunomodulatory Therapies in Patients With NMD

Some types of NMD are associated with immune-mediated pathogenesis. Patients with NMD who receive immunomodulatory therapy (IMT) are likely at increased risk of having more severe COVID-19 infections (5). Recently, a consensus statement on IMT management during the COVID-19 pandemic is emerging to guide patients and clinicians (5, 6). Based on the pandemic burden of the region, patient compliance and caregiver support, dose reduction of certain IMTs, or switching to alternative agents for high-risk NMD patients can be considered. The decision to temporarily suspend, reduce, or change IMT should be discussed with NMD experts, and patients should not proceed without consultation (9).

Notably, sudden discontinuation of corticosteroids may induce a flare-up of the underlying disease, requiring a higher stress dose and increasing hospitalization risk. Especially during acute illness or hospitalization related to COVID-19, it may be necessary to increase the steroid dose (amount or frequency) and follow the recommended dose in the infection/stress guidance to avoid hyposurrenalism (10, 11). Otherwise, there is no evidence suggesting that intravenous immunoglobulin (IVIG), therapeutic plasmapheresis, or complement inhibitor (Fc receptor antagonists, e.g., efgatirgimod) can increase the risk of COVID-19 infection or aggravate the disease severity (2, 6).

Some cases with severe COVID-19 infection may be related to a cascade of immune dysregulation and overreaction of inflammatory pathways (12). Therefore, certain immunomodulatory drugs used in the treatment of NMD may help resist SARS-CoV-2 infection or ameliorate severe complications. For instance, hydrocortisone and dexamethasone are reported to potentially benefit treating COVID-19 patients with severe cardiopulmonary complications (13). Eculizumab, a monoclonal antibody against complement, has recently been investigated as a potential treatment in autoimmune MG (14). Moreover, the treatment of severe COVID-19 with eculizumab is currently undergoing a clinical trial (NCT04288713) (15).

### Adjustment of Disease-Modifying Therapies in Patients With NMD

Hospitalization should be reserved for emergencies. However, preventive strategy with the requirement of isolation may affect treatment options requiring in-hospital setting for administration, such as infusion of nusinersen (Spinraza®), glucosidase alfa (Myozyme®), rituximab, and IVIG. These treatments should not be discontinued arbitrarily, but consideration should be given to shifting treatment to a nonhospital setting (home-visiting or outreach nurse), and collaboration with the pharmaceutical company can be negotiated.

It is recommended to continue intrathecal injections as much as possible for infants with type 1 SMA and children with type 2 SMA. As per the manufacturer's recommendation, the half-life of nusinersen is more than 100 days, affecting alternative splicing for several months (16). Therefore, if these SMA patients miss the planned dose after 4 months, they should be given a subsequent dose of nusinersen on the date minus the number of missed days originally scheduled to ensure a sufficient restoration of SMN protein (17). However, for adolescents and adults, injections could be delayed by 1–4 months, depending on the clinical progression (8). Inspiringly, the U.S. Food and Drug Administration (FDA) has recently approved risdiplam (Evrysdi®) as the first oral and at-home treatment for patients with all types of SMA (18). This therapeutic agent may provide a flexible alternative to SMA-modifying therapy, especially during the pandemic.

Suspending enzyme replacement therapy for 1–3 months is unlikely to cause serious deterioration of the disease. However, there is limited evidence to accurately estimate the risk after a relatively short interruption of treatment (19). It is recommended that patients with Duchenne muscular dystrophy (DMD) continue to use drugs to prevent or treat cardiomyopathy, such as angiotensin-converting enzyme inhibitors and/or angiotensin receptor blockers (10). IVIG can be changed to subcutaneous immunoglobulin whenever possible (2). The benefits of transitioning from a hospital-based center to at-home infusion should be weighed and may depend on the patient's overall COVID-19 risk, transportation requirement, geographic resources, and insurance coverage. The treatment efficacies between hospital and home facilities are still being studied. Besides, trial centers should be consulted for advice on clinical trials.

### Modification of Providing Physiotherapy for Patients With NMD

The pandemic has also prompted the reallocation of rehabilitation services. For many patients with NMD, it is crucial to maintain joint flexibility, muscle strength, and endurance even during a pandemic; therefore, rehabilitation advice should be obtained through alternative strategies, including telemedicine (8). Considering telerehabilitation for NMD patients, the evidence-based database of Cochrane review lacks a comprehensive analysis for these patients. Nevertheless, a retrospective study reported that providing rehabilitation for 26 patients with mixed NMD through telemedicine improved their cognition, self-care, quality of life, and motor function (20). Since most NMD referral hospitals and treatment centers have kept essential telehealth activities, patients and their families are encouraged to contact these departments to obtain personalized support (telerehabilitation) (2, 6). Self-rehabilitation and exercises can be set up according to age, current motor function, and personal goals. As telemedicine's most encountered limitation is the knowledge gap between the professional providers and home-based caregivers, these programs should be deliberately simple and guided to be delivered by caregivers who are not health professionals. Importantly, effective telemedicine services rather than physical contact services can significantly reduce the risk of infection and spread of COVID-19. Telemedicine approaches can include but are not limited to applying novel technologies such as e-mail, instant messaging applications, and hands-free telephone or webcam interviews. Other innovative communication platforms are emerging and quickly spread to the medical field (21).

## Management of NMD Patients With Covid-19 Infection

### Respiratory Support for NMD Patients With COVID-19 Pneumonia

As respiratory involvements lead to the most deaths and morbidities of patients with NMD, recent evidence indicates accumulating exacerbation risks caused by COVID-19 in NMD patient group (2, 6). Especially in some types of NMD, patients who have respiratory muscle involvement and/or cardiomyopathy are likely at greater risk of contracting a severe COVID-19-related complication.

At this time, respiratory care in NMD patients requires a deliberate revision during the COVID-19 pandemic (22). The WHO recommends that all COVID-19 patients with respiratory distress or hypoxemia be supported immediately with oxygen supplement at 5 L/min and that flow rates are titrated to attain SpO_2_ ≥ 90% in nonpregnant adults and SpO_2_ ≥ 92–95% in pregnant patients (23). Otherwise, hypercapnia is not a typical feature of SARS-CoV-2 pneumonia, and its presence may implicate the deterioration of respiratory pump weakness (24), which may progress more rapidly in NMD patients. Thus, NMD patients with COVID-19 pneumonia should be closely monitored, such as increased oxygen demand, progressive CO_2_ retention, and acidosis. NMD patients presenting with interstitial pneumonia should consider early ventilation support. It should be kept in mind that hypoxemia complicated by COVID-19 pneumonia may even rapidly cause pump failure in previously compensated patients, while hypercapnia can further aggravate the disease process (6).

It has been proposed that COVID-19-related acute respiratory distress syndrome (CARDS) is distinct from the typical form of acute respiratory distress syndrome (ARDS) (25). ARDS usually does not respond to solitary oxygen therapy because hypoxemic persistence is typically the result of intrapulmonary ventilation-perfusion mismatch or shunt. In contrast, CARDS is characterized by relatively high lung compliance in the intermediate stage of COVID-19 pneumonia but significantly reduced in the later stage (25). Therefore, the treatment strategy initiated in CARDS has now shifted to the early support of noninvasive ventilation (NIV) instead of intubation and mechanical ventilation (26). However, data on patients with Middle East respiratory syndrome (MERS) indicate a high failure rate of management with NIV (27), whether a similar outcome in patients with SARS-CoV-2 infection is still unclear. In several large cohorts of COVID-19 patients admitted to the ICU due to acute respiratory failure (ARF), NIV was used in 11–62% of patients, compared with 30–88% of endotracheal intubation with invasive mechanical ventilation (6). Especially in NMD patients, applying NIV as a first-line intervention for ARF has been widely advocated for its potential benefits such as shorter ICU stays and improved overall survival and to avoid intubation and facilitate extubation (28, 29). In addition to respiratory support, the purpose of restoring the pulmonary function should also include treatment strategies for COVID-19-related cytokine storms (30).

However, emerging studies have limited NIV use in severe cases of COVID-19 pneumonia due to a concern that NIV may bring the risk of widespread exhaled airborne virus (31). It may be explained that a single circuit with only one hose is always equipped in an NIV set; therefore, the exhaled gas is not filtered through a valve. Thus, NIV with high airflow may result in more aerosolized COVID-19 virus spreading than conventional ventilators. Recent reports show that modified systems with appropriate interface fitting might reduce viral contamination in the healthcare environment (32). These modified strategies may include the following: (1) before starting or stopping NIV, the patient's mask must be worn tightly, and caregivers must wear personal protective equipment; and (2) a full-face mask for NIV is preferred and should be sealed as tightly as possible. An antiviral filter should be used at the ventilator outlet of the inhalation circuit and after the mask (8). Several innovative NIV interface designs have been applied clinically, providing a more closed ventilation system (22, 26, 33). It should be addressed that patients undergoing NIV should remain under close monitoring and shift to a conventional ventilator if showing rapid deterioration or lack of improvement (6). Intubation may be necessary upon progressive deterioration during COVID-19 infection. However, patients with end-stage NMD, such as ALS and DMD, may request conservative approaches without aggressive management. In this case, an in-depth discussion of palliative care can begin.

In addition, the risk of anesthesia in patients with NMD varies greatly because it depends mainly on baseline lung function and the presence of comorbidities (34). In some NMD cases, masticatory muscle atrophy and limited cervical spine mobility may complicate the intubation process. Therefore, intubation in NMD patients should always follow the guidelines for difficult airway management (34). Besides, patients with NMD should be cautious about the side effects of neuromuscular blockers and anesthetics. Succinylcholine, a depolarizing muscle relaxant, should be avoided in patients with muscular dystrophies, motor neuron diseases, and intrinsic muscle disease because of the risk of malignant hyperthermia, fatal hyperkalemia, and rhabdomyolysis (35–37). Nondepolarizing muscle relaxants should be reduced dosage and titrated carefully in some categories of NMD, including myotonic muscular dystrophy, MG, congenital myasthenic syndrome, SMA, polymyositis, and dysimmune neuropathies (36, 37). In addition, due to the advantages of easy controlled dosage and shorter onset time, intravenous anesthetics are preferable to volatile agents in most patients of NMD (37).

Intermittent prone positioning during the mechanical ventilation support seems beneficial to improve the oxygenation of patients with COVID-complicated ARDS (38). However, this position might be contraindicated in NMD patients. NMD patients with severe kyphoscoliosis may compress the tracheobronchial tract against the vertebral body on prone positioning (39). NMD patients with complicated deformities in anatomy may affect the choice of pulmonologists for prolonged prone positioning during the ventilator support.

### Effects of COVID-19 Therapeutic Agents on Patients With NMD

Emerging therapeutic trials have been initiated in the context of COVID-19 infections. Although several preliminary data of clinical trials appear promising, the evidence on infected NMD patients is limited. Certain investigational treatments for COVID-19 may be prescribed for NMD patients compassionately, as outside trial conditions. Nevertheless, this off-label medication use in NMD patients should only be taken after consultation with NMD specialists (2, 8). Table 2 summarizes the specific precautions for NMD patients when receiving drugs prescribed to treat COVID-19.

**Table 2:** COVID-19 therapeutic agents with potential NMD complications.

**Therapeutic agents**	**Potential NMD-relevant side effects**	**NMD patients with particular caution in use**
Hydroxychloroquine	•QTc interval prolongation may lead to cardiac arrest secondary to cardiac arrhythmia, especially when combined with other QTc-prolonging drugs •Newly onset or exacerbation of MG •Risk of toxic neuropathy and myopathy •Cardiotoxicity and cardiomyopathy •Elevated serum CK level	•Autoimmune and congenital MG •NMD with myocardial involvement, i.e. DMD/BMD •Andersen-Tawil syndrome (a rare form of periodic paralysis) •Myotonic dystrophy
Azithromycin	•QTc interval prolongation •Risk of worsening MG	NMD patients who have similar susceptibility to hydroxychloroquine
Lopinavir/Ritonavir	•QTc interval prolongation may lead to cardiac arrest secondary to cardiac arrhythmia, especially when combined with other QTc-prolonging drugs •Risk of toxic myopathy with rhabdomyolysis, especially in combination with a statin	Careful monitoring of serum CK levels when treating in myopathic patients
Remdesivir	•Myalgias in healthy controls •Elevation in liver enzymes	All kinds of susceptible NMD patients should be monitor serum liver enzymes and CK level
Eculizumab	•Myalgias and arthralgias •Elevation in liver enzymes	All kinds of susceptible NMD patients should be monitor serum liver enzymes and CK level

*NMD, neuromuscular disorder; CK, creatinine kinase; MG, myasthenia gravis; DMD, Duchenne muscular dystrophy; BMD, Becker muscular dystrophy*.

Chloroquine and its less toxic derivative hydroxychloroquine have been indicated for antimalaria and some chronic rheumatic diseases. Recent reports have raised concerns about the possible beneficial effects of hydroxychloroquine on SARS-CoV-2, and it has been tested as a supplementary therapeutic agent in hospitalized COVID-19 patients (40). Nevertheless, the efficacy of hydroxychloroquine against COVID-19 is not yet clear. Some small uncontrolled studies have shown benefits, and at least one controlled research has shown the opposite finding (41–43).

In patients with NMD, particular attention must be paid to hydroxychloroquine treatments for COVID-19 due to possible adverse effects. The most potentially dangerous complication of hydroxychloroquine use is arrhythmias, especially QTc prolongation (44). The risk is increased when hydroxychloroquine is combined with azithromycin. Great attention must be given to certain patients who have NMD-related cardiac involvement (45). Particularly during the long-term hydroxychloroquine use, an increased risk of conductive disorders (QTc prolongation) and myocardial damage can potentially worsen systolic left ventricular dysfunction in patients with certain types of NMD (2, 5, 8). Currently, hydroxychloroquine is suggested contraindicated to patients with DMD or myotonic dystrophy for treatment options of COVID-19 (10). Besides hydroxychloroquine, some drugs now used against COVID, such as lopinavir and ritonavir, also contribute to the QTc prolongation (46).

Notably, hydroxychloroquine, especially in combination with azithromycin, can cause new onset or aggravation of MG (5, 47). Increased creatine kinase (CK), vacuolar myopathy, and toxic neuropathy may occur in some patients with long-term hydroxychloroquine use (6, 48). Complications of toxic myopathy with rhabdomyolysis in patients treated with lopinavir/ritonavir combined with a statin have been reported (49). However, COVID-19 may be complicated with myositis presenting myalgia or fatigue and increased CK in about one-third of admitted patients (50, 51). It is recommended to conduct careful risk/benefit assessment before dosing these agents on patients with myopathy, and regular monitoring of serum CK levels is required when receiving these drugs (5). It is currently not recommended to use these drugs for prophylaxis purposes in NMD patients because their preventive efficacy has not been proven and may cause serious toxicity.

## Conclusion

COVID-19 is a rapidly evolving field, and the evidence-based best practices in NMD patients are subject to revision frequently. Patients with NMD present unique management challenges in the COVID-19 pandemic. The severity of manifestations and potential complications vary with individual circumstances and patients. Individually designed care plans coordinated among multiple providers are critical to optimizing the treatment effects of these vulnerable patients. Since the possibility of second waves of the pandemic, we will need a robust reorganization of neuromuscular centers, where the role of telehealth providers will be significant. Collaborative efforts among institutions in the NMD community will help provide the data to inform the modified management of NMD patients infected by COVID-19. Importantly, close collaboration must be integrated into a multidisciplinary care team, including but not limited to neuromuscular specialists, intensive care specialists, pulmonologists, rehabilitation therapists, and gastroenterologists. These teams should support their hospital to define standardized and targeted care for NMD patients during the COVID-19 pandemic.

## Author Contributions

Y-HT and T-HC contributed to the conception and design of the study, acquisition of data, revision of the manuscript critically for relevant intellectual content, and final approval of the version to be published. All authors contributed to the article and approved the submitted version.

## Conflict of Interest

The authors declare that the research was conducted in the absence of any commercial or financial relationships that could be construed as a potential conflict of interest.
